# *Drosophila melanogaster* Response to Feeding with Neomycin-Based Medium Expressed in Fluctuating Asymmetry

**DOI:** 10.3390/insects11060378

**Published:** 2020-06-18

**Authors:** Kemal Büyükgüzel, Ender Büyükgüzel, Ewa Chudzińska, Anetta Lewandowska-Wosik, Renata Gaj, Zbigniew Adamski

**Affiliations:** 1 Department of Biology, Faculty of Arts and Science, Zonguldak Bülent Ecevit University, 67100 Zonguldak, Turkey; buyukguzelk@hotmail.com; 2 Department of Molecular Biology and Genetics, Faculty of Arts and Science, Zonguldak Bülent Ecevit University, 67100 Zonguldak, Turkey; endericen@hotmail.com; 3 Department of Genetics, Faculty of Biology, Adam Mickiewicz University, 61-614 Poznań, Poland; ewa.chudzinska@amu.edu.pl (E.C.); anelew@amu.edu.pl (A.L.-W.); 4 Department of Agricultural Chemistry & Environmental Biogeochemistry, Poznan University of Life Science, 60-637 Poznań, Poland; renata.gaj@up.poznan.pl; 5 Electron and Confocal Microscope Laboratory, Faculty of Biology, Adam Mickiewicz University, 61-614 Poznań, Poland; 6 Department of Animal Physiology and Development, Faculty of Biology, Adam Mickiewicz University, 61-614 Poznań, Poland

**Keywords:** *Drosophila melanogaster*, neomycin, fluctuating asymmetry

## Abstract

The fruit fly *Drosophila melanogaster* is a model species used for a wide range of studies. Contamination of *Drosophila* cultures with bacterial infection is common and is readily eradicated by antibiotics. Neomycin antibiotics can cause stress to *D. melanogaster*’s larvae and imagoes, which may affect the interpretation of the results of research using culture from neomycin-based medium. In the present study, fluctuating asymmetry (FA), one of the important bioindicators of stress, was measured. Larvae and imagoes of a wild-type *D. melanogaster* strain were exposed to various concentrations of neomycin. The size of anal papillae and selected wing veins were measured using scanning electron and light microscopy, respectively. Next, the FA was checked. The values obtained for larval anal papillae appeared to be concentration-dependant; the FA indices increased with the concentration of neomycin. The wing FA presented a large but variable correlation, depending on the measured vein. However, the mean length of veins was the highest for the control group, with neomycin-exposed groups showing lower values. The research showed that neomycin may cause sublethal stress in *D. melanogaster*, which manifests in increased FA indices. This suggests that neomycin can cause physiological and developmental stress in insects, which should be taken into account when interpreting the results of studies using these model organisms.

## 1. Introduction

The fruit fly *Drosophila melanogaster* (Meigen) (Diptera: Drosophilidae) is a commonly used model for a wide range of studies, because of its short life cycle, adaptation to different ecological conditions, and ease of rearing in the laboratory. Previous experiments have demonstrated that different concentrations of some organophosphorus insecticides, plant secondary metabolites, and food dyes have toxic effects on survivorship of *D. melanogaster* [1,2,3,4]. Antibiotics that possess antibacterial, antifungal, and anthelmintic properties have been incorporated into insect-rearing media for decades. Their influence on life parameters and their effective concentrations for control of microbial contamination in diets have been studied for many species [5,6,7,8,9,10]. It has been shown, for example, that clinically significant doses of some antibiotics lead to mitochondrial dysfunction and oxidative damage in patients caused by reactive oxygen species (ROS) [11]. This raises the possibility that antibiotics incorporated into diets may also exert damaging effects on insects or decrease quality of the products of their activity, like honey [12].

Numerous studies have indicated that the use of antimicrobial substances that prevent mold and bacteria from contaminating insect cultures can lead to developmental retardation, decreased survival, and short longevity of certain insects [6,7,9,13,14,15,16,17]. Many insect species are routinely maintained in very large colonies for research purposes and for large-scale insect pest management. The potential influence of dietary antibiotics on insect biology is of importance because their use may increase the costs of maintaining insect cultures, affect insect physiology, and potentially reduce the biological performance of insects reared for research and biological control programs. In the case of *D. melanogaster*, it may also lead to misinterpretation of the results of experiments carried out using this model organism.

One of the antibiotics used in insect breeding is neomycin aminoglycoside—a broad-spectrum substance, naturally produced by *Streptomyces fradiae*, often found in the environment [18]. This antibiotic inhibits protein synthesis at initiation and elongation stages by binding 30S subunits, and sometimes 50S subunits, of ribosomes in prokaryotes [19,20]. There is limited information on the effects of neomycin on insects, whereas its physiological, biochemical, and molecular effects on the tissues and organs of higher eukaryotic organisms, including humans and animals, are well-known [21,22,23,24]. It was shown that higher levels of neomycin in both in vivo and in vitro treatments disorganized polytene chromosomes in the salivary glands of dipteran fourth instar *Chironomus* sp. larvae, suggesting that neomycin may affect gene activity in insects [25]. Based on general pro-oxidative effects, we demonstrated that antibiotics and anthelmintic exert oxidative damage and stimulate increased antioxidant and detoxification enzymes in some model insects [26,27]. Like other antibiotics, neomycin can also have unintended effects on animals and microorganisms in the environment when it is released through various uncontrolled routes [18,28]. This means that in addition to laboratory-controlled exposure to neomycin, *Drosophila* may also be exposed to this antibiotic under natural conditions. Like all insects, fruit flies must adapt to the presence of different substances in the environment. Badyaev [29] indicated that stressful and unsuitable environments may impair the integration of morphological regulatory systems, causing alterations of normal development. In this context, morphology provides an excellent opportunity to investigate the factors that may induce changes during growth. There is no information on the effects of antibiotics and other antibacterial substances on the developmental stability of insects, including *Drosophila* spp.

Does adding neomycin to the medium cause developmental instability in *Drosophila*? If so, can this be demonstrated by analyzing the index of fluctuation asymmetry? We predicted that more asymmetrical individuals with developmental instability might arise when insect larvae are reared on a diet containing antibiotics, in terms of nutritional stress, as observed for species that are forced to grow in an unfavorable habitat. Such asymmetry has been reported as a sign of environmental stress by several researchers [30,31]. In this paper, we measured fluctuating asymmetry (FA) in the size of anal papillae of larvae, as well as in wing-vein length of imagoes, to examine the hypothesis that breeding insects with a neomycin-containing diet under laboratory condition may create a stressful environment for the *D. melanogaster* causing developmental instability. FA is often treated as a general biomarker of environmental and/or genetic stress [32,33,34]. Anal papillae are clearly distinguishable structures of dipteran larvae. They are important structures for the regulation of osmotic conditions, due to their modified epidermis, e.g., lack of exocuticle and modifications of the size and ultrastructure of epithelial cells [35]. Therefore, they can be influenced by various factors and may be early markers of stress. They can be easily observed and measured, and, to the best of our knowledge, the FA index has not been determined in *D. melanogaster* larvae using these structures to date. Various studies have shown differences in the morphology of wings reflected in the FA are sensitive indicators of environmental stress [36,37,38]. Moreover, Debat et al. [36] indicated that wing shape can vary in more flexible ways and be more informative than simple traits such as the size of other body parts when investigating deteriorated larval survival, development, and adult fitness of insects exposed to environmental stressors (temperature, insecticides, and other chemical pollutants, dietary additives such as antimicrobial agents). Since the method of FA analysis is easy to apply, fast, and relatively cheap, it can be routinely used to compare numerous potential stressors on various species, worldwide.

This method was therefore applied to determine the possible stressful effects of sublethal concentrations of neomycin on *D. melanogaster* after rearing larvae on diets amended with the aminoglycoside antibiotic neomycin.

## 2. Materials and Methods

### 2.1. Drosophila melanogaster Culture

Wild-type (Oregon R strain) *D. melanogaster* from a laboratory culture was used in this study. The experiments were carried out under laboratory conditions. The stock culture was xenically maintained by rearing first instars to adults on an artificial diet [39,40]. The colony was maintained and the tests were conducted at 25 ± 2 °C, 60–70% relative humidity, and on a photoperiod of 12:12 (L:D) h. Newly emerged adults were used to maintain the stock culture. Insects were reared in 15 mL vials (26.5 × 58.7 mm, Yıldız Chemistry Co., Ankara, Turkey) with ~5 mL of artificial diet. The standard diet was composed of 8 g of agar–agar ultrapure (Merck, Darmstadt, Germany), 20 g of sucrose (BioUltra, 99 %, Sigma Chemical Co., St. Louis, MO, USA), 11.78 g of dried powder yeast (Dr. Oetker Food Co., Torbalı-İzmir, Turkey), 0.8 g of ascorbic acid (BioUltra, 99%, Sigma), 7.72 mL of nipagine (SigmaUltra, p-hydroxybenzoic acid methyl ester, crystal), 36 g of mashed potatoes (Knorr, Unilever Co., Ümraniye, İstanbul, Turkey), and 1000 mL of distilled water. Diet in liquid form was poured into the individual vials, and the vials were maintained at room temperature for 30 min to allow the diet to solidify. Ten or 15 males and females of the same age were placed in individual vials and maintained for 24 h, the time necessary for fertilization and oviposition. Vials were plugged with cotton to prevent escape of the flies and to ensure adequate ventilation. After 24 h, adults were transferred into new vials with the control and amended diets according to treatment group as per Reis [41]. Transfers occurred every 3 days. Methods used to prepare and dispense diets and the rearing of instars are described in detail in Roberts [42].

### 2.2. Feeding Experiments

Neomycin sulfate (white powder form, neomycin *O*-2,6-diamino-2,6-dideoksi-α-d-glukopiranozil-(1-3)-*O*-β-d-ribofuranozil-(1-5)-*O*-[2,6-diamino-2,6-dideoksi-α–d-glukopiranozil-(1-4)]-2-deoksi-d-streptamin, sulfate (trisulfate salt, aqueous) water-soluble in white powder form 90–95%, Sigma Chemical Co., St. Louis, MO, USA) was directly incorporated into diets at four different concentrations of 150, 300, 600, and 900 mg/L diet while the diets were in the liquid state. These concentrations were based on the results of previous experiments with sodium tetraborate and boron derivatives studied using *D. melanogaster* [43,44] and other insects [7,45], because it allowed fruit flies to complete their development to the adult stage in pre-feeding tests. Control diets had no neomycin sulfate. Neonates were placed into separate vials using a fine brush and reared until adult emergence on the artificial diets amended with given concentrations of neomycin, which took about 10 days. Newly emerged third instar larvae and adults from each assay were selected for fluctuating asymmetry (FA) observations.

### 2.3. FA Measurement

#### 2.3.1. Larvae

**Figure 1:**
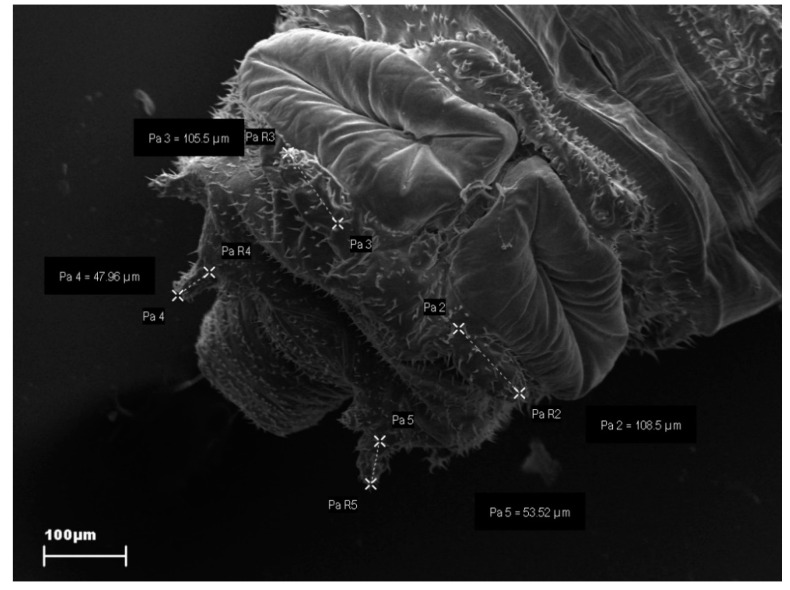
SEM picture of measured anal papillae. *Drosophila melanogaster* larvae exposed to 300 mg neomycin/L.

Due to the size of the larval anal papillae and their location, scanning electron microscopy (SEM) was used to measure them. Larvae were fixed in 2% glutaraldehyde, buffered with 0.1 M sodium cacodylate (pH 7.2) for 2 h, and then postfixed in 1% osmium tetroxide and dehydrated through a series of ethanol–water solutions of increasing concentration. Samples were critical-point dried, coated with gold, and observed using a Zeiss Evo 40 SEM (Figure 1).

For larvae, the length of the two anal papillae was measured twice (left and right appendix were distinguished) by two independent people, and the absolute difference in the length of two appendixes generated similar results (Table 1), indicating high repeatability. The FA index was defined as an absolute difference between the right and left appendixes, standardized by the mean. The presence of asymmetry was confirmed using an ANOVA test (Table 2), which tested the differences in length between the longer and shorter appendix in a pair, as previously described [46,47].

#### 2.3.2. Imagoes

**Figure 2:**
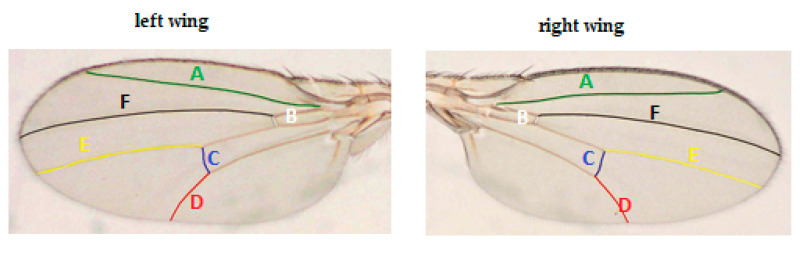
The six measured veins (symbols from A to F) of *Drosophila melanogaster*.

Wild-type *Drosophila melanogaster* imagoes from each assay were selected. FA index was defined as the absolute difference between the right wing and the left wing veins standardized by the mean. The length of six veins was measured under the microscope (4× magnification), using the CellB program (Olympus Co.) (Figure 2) as the base to compute the FA indices.

The length of veins (from the left and the right wings) was measured twice by two independent people, and the absolute difference in the length of two appendixes generated similar results (Table 3), indicating high repeatability.

The presence of asymmetry was confirmed in three veins, B, C, and D, by the ANOVA test (Table 4), which tested the differences in length between longer and shorter veins in the pairs [46,47]. Therefore Veins A, E, and F were not included in further analyses.

### 2.4. Biometric and Statistical Analyses

The type of asymmetry of the two anal papillae was revealed by testing the normality of the right-minus-left value distribution using the Shapiro–Wilk test, which detected the absence of anti-symmetry (1: *p* = 0.56, 2: *p* = 0.71). Anti-symmetry and trait size were checked according to the protocol described by Kozlov and Niemelä [46]. Absence of directional asymmetry was confirmed by the zero mean value (1: *p* = 0.46, 2: *p* = 0.58). Thus, observed asymmetry was classified as FA.

For imagoes, the type of asymmetry was revealed by testing the normality of right-minus-left values distribution using the Shapiro–Wilk test, which detected the absence of anti-symmetry (B: *p* = 0.80, C: *p* = 0.62, D: *p* = 0.97). Anti-symmetry and trait size were checked according to the protocol described by Kozlov and Niemelä [46]. Absence of directional asymmetry was confirmed by the zero mean value (B: *p* = 0.46, C: *p* = 0.35, D: *p* = 0.58). Thus, observed asymmetry was classified as FA. The Shapiro–Wilk test showed a normal distribution of the FA indices. The descriptive statistics of arithmetic mean, standard deviation, range, and the coefficient of variability were calculated. Next, the Student *t*-test was conducted to check statistically significant differences in terms of the fluctuating asymmetry factor in the studied dose of neomycin [48]. To provide a graphical presentation of results, a principal-component analysis (PCA) and cluster analysis were conducted.

Biometric data were statistically analyzed using StatSoft Statistica (StatSoft Inc., Tulsa, OK, USA).

## 3. Results

### 3.1. Results of Larvae Fluctuation Asymmetry Measurements

**Figure 3:**
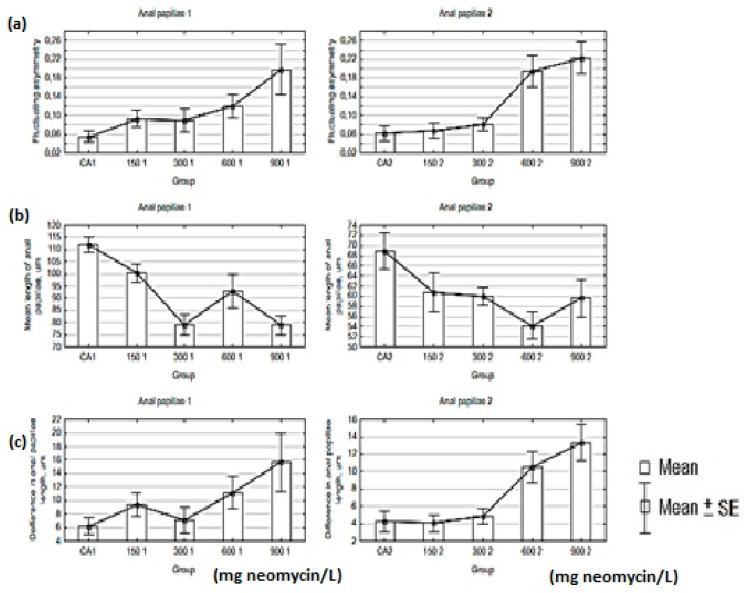
Graph of mean and standard error (SE) of fluctuating asymmetry (**a**), mean length of anal papillae (**b**), and differences in anal papilla length (**c**) for the two studied appendixes.

The value of FA ranged from 0.055 to 0.198 for the first anal papillae and from 0.061 to 0.223 for the second ones. For both appendixes, the highest FA value was in the group treated with a concentration of 900 mg/L, and the lowest was in the control group (Table 5, Figure 3). The mean length of both appendixes was the highest in the control group (CA). The lowest value for the first anal papillae was in the groups treated with concentrations of 300 mg/L and 900 mg/L of neomycin, and for the second anal papillae the lowest value was observed in the group treated with a 600 mg/L dose of antibiotic. The highest values of differences in length of anal papillae for both appendixes were in the 900 mg/L neomycin group, and the lowest value was for the control group (Figure 3).

**Figure 4:**
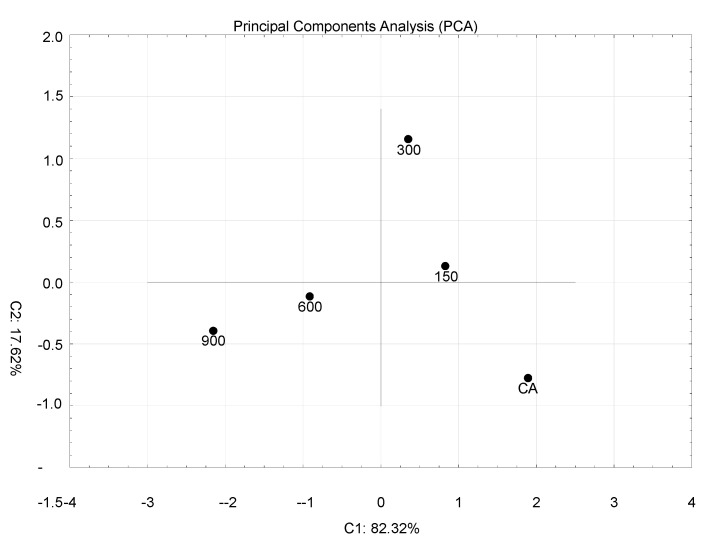
GGraph of principal-component analysis for larva anal papillae.

**Figure 5:**
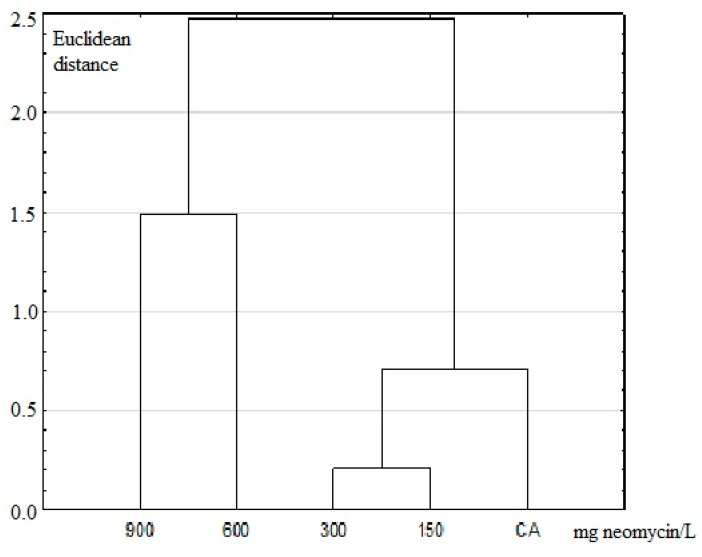
UPGMA dendrogram based on two anal papillae: 900–150 doses of neomycin (150–900 mg/L), CA—the control group.

**Figure 6:**
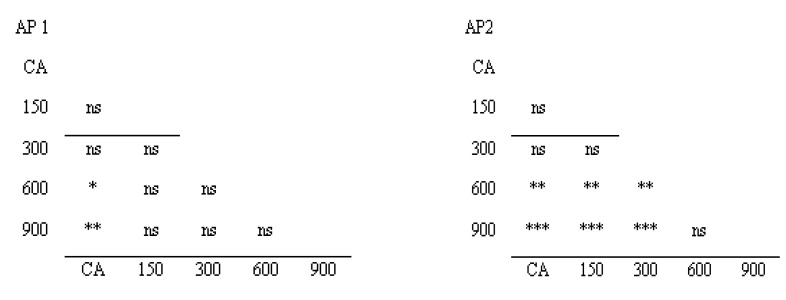
Student t-test applied between studied doses of neomycin (150–900 mg/L) and control group (CA) for two studied appendixes. AP1—the first pair of anal papillae, AP2—the second pair of anal papillae, * *p* < 0.05, ** *p* < 0.01, *** *p* < 0.001, ns—*p* > 0.05.

Principal-component analysis (PCA) (Figure 4) and unweighted pair group method with arithmetic mean (UPGMA) dendrogram (Figure 5) made using FA indices based on the two anal papillae showed that the 150 mg/L and 300 mg/L treatments formed one group, the second group included doses of neomycin 600 mg/L and 900 mg/L, and the control group was distinct. The Student *t*-test was conducted to check statistically significant differences in terms of the fluctuating asymmetry index in the studied dose of neomycin. In both appendixes, the control group differed statistically from 600 mg/L and 900 mg/L of neomycin treatments. For the second anal papillae, the groups treated with concentrations of 150 mg/L and 300 mg/L of neomycin differed statistically from groups treated with 600 mg/L and 900 mg/L of neomycin (Figure 6).

## 3.2. Results of Imago Fluctuation Asymmetry Measurements

**Figure 7:**
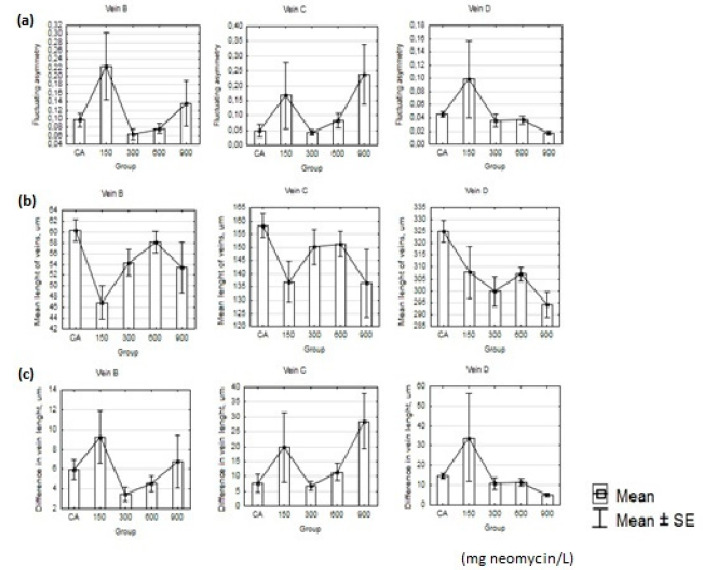
Graph of mean and standard error (SE) of fluctuating asymmetry (**a**), mean length of veins (**b**), and differences in vein length (**c**) for three studied veins.

The value of FA ranged from 0.063 to 0.224 for Vein B, from 0.044 to 0.237 for Vein C, and from 0.017 to 0.099 for Vein D. For Vein B, the highest FA value was for the 150 mg/L neomycin treatment and the lowest was for the 300 mg/L treatment. For Vein C, the highest FA value was for the 900 mg/L treatment and the lowest was for the 300 mg/L treatment. For Vein D, the highest FA value was for the 150 mg/L treatment and the lowest was for the 900 mg/L treatment (Table 6, Figure 7). The mean lengths of Veins B and C were the highest in the control group (CA) and the lowest in the 150 mg/L neomycin treatment group. The mean length of Vein D was also the highest in the control group (CA) but the lowest in the 900 mg/L neomycin treatment group. For all veins, the CA group had the longest veins, and the shortest veins were in the 150 mg/L treatment group for Veins B and C, whereas for Vein D the shortest veins were in the 900 mg/L treatment group. The highest values of differences in vein length for Veins B and D were for the concentration of 150 mg/L neomycin, and for Vein C were in the 900 mg/L treatment. The lowest value of differences was in the 300 mg/L group for Veins B and C, and in the 900 mg/L group for Vein D.

**Figure 8:**
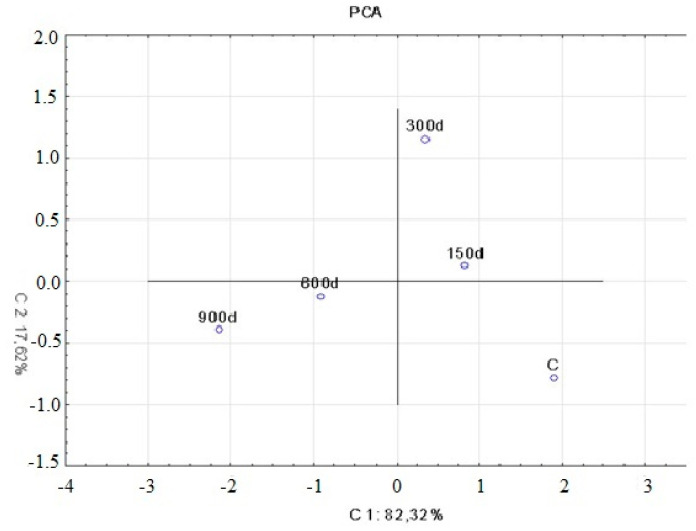
Graph of principal-component analysis for imagoes.

**Figure 9:**
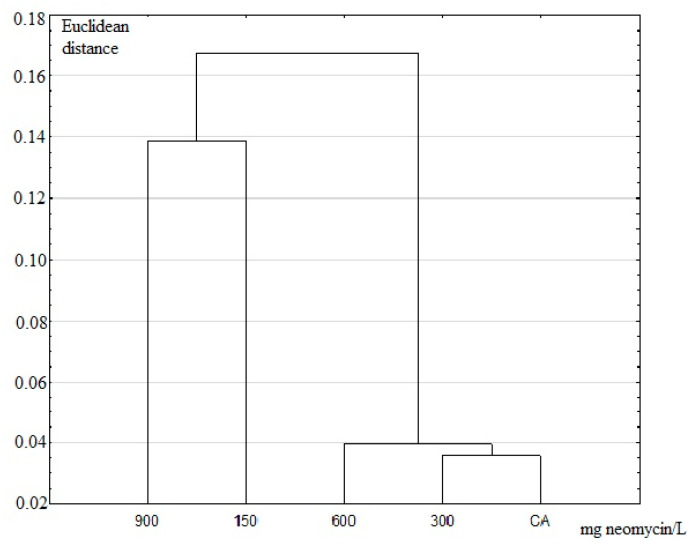
UPGMA dendrogram based on three studied veins. 900–150 doses of neomycin (150–900 mg/L), CA—the control group.

Principal-component analysis (PCA) (Figure 8) and UPGMA dendrogram (Figure 9) made using the FA indices based on the three veins showed that the CA, 300 mg/L, and 600 mg/L doses formed one group, and the 150 mg/L and 900 mg/L doses of neomycin were distinct from this group and from each other.

**Figure 10:**
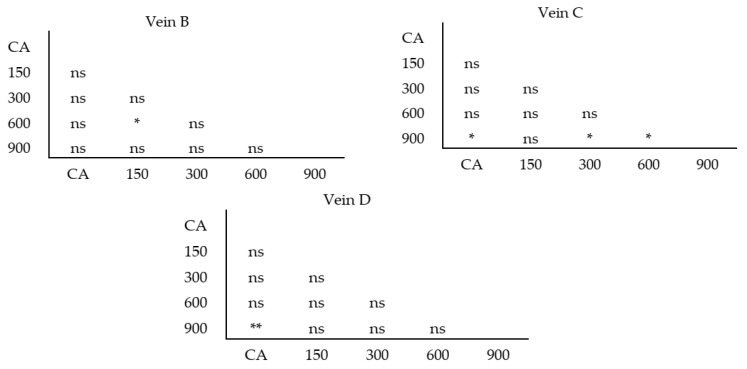
Student *t-*test between studied doses of neomycin (150–900 mg/L) and control group (CA) for three studied veins; * *p* < 0.05, ** *p* < 0.01, ns—*p* > 0.05.

The Student *t*-test was conducted to check statistically significant differences in terms of the fluctuating asymmetry factor in the studied concentrations of neomycin. The 150 mg/L concentration neomycin treatment differed from the 600 mg/L treatment with regard to Vein B. The 900 mg/L concentration differed from the 300 mg/L, 600 mg/L, and CA treatments with regard to Vein B, and because Vein D in the 900 mg/L treatment differed from that in CA (Figure 10).

## 4. Discussion

Fluctuating asymmetry (FA) is a measure of developmental instability expressed as a difference in accurate bilateral symmetry, caused by environmental stresses, and genetic problems during development. Environmental stressors that may induce FA in insects, usually include temperature extremes, oxygen deficiency, poor diet, and chemical pollution [49,50,51,52]. There is no information on the effects of antimicrobial substances such as antibacterials, antivirals, and antihelminthics on the fluctuating asymmetry (FA) of insects, including *D. melanogaster*. In many studies, the effect of various pesticides on the FA of numerous insect groups has been investigated [3,4,49,50,51,52]. Antipin and Imasheva [52] examined the effects of a chlorine–organic insecticide, endosulfane (thiodan), on phenotypic and genetic variation in wing length, thorax length, the number of orbital bristles, and the number of sternopleural bristles of *D. melanogaster*. Experiments carried out on *Ceriagrion* sp. larvae suggest that FA may be regarded as an indicator of pesticide stress. [50]. The usefulness of FA has also been demonstrated by investigating the effect of temperature and pesticide exposure on larval development in *Copera annulata* (Selys), among other studies. Insecticide treatment did not significantly affect the mortality of the larvae of damselfly, but the FA values of three traits decreased at lower concentrations, and then increased slowly with increased insecticide doses [49]. The results of these studies indicated that traits related to body size, expressed by the FA index, could be used as a suitable universal indicator of the environmental stress caused by chemical pollutants in populations of insects. A previous study reported the effects of chemicals such as arsenic and lead on FA in *Drosophila* [53]. It is known that nutrients available in the larval period are effective in all developmental stages of the insect [54]. For this purpose, the content and quality of artificial diets used for growing insects in the laboratory environment must be balanced [55,56,57,58,59]. Various antifungal, antibacterial, and antihelminthic agents are frequently used to improve diet quality. In this study, we used an asymmetry indicator to determine whether neomycin impaired the nutritional quality of the diet and led to nutritional stress. We checked whether the relationship between asymmetry parameters and the quality of the artificial diet could be determined. The effect of nutritional stress during development on FA has been addressed in a range of organisms [60,61]. Vijendravarma and coworkers (2011b) observed that larval malnutrition of *Drosophila melanogaster* had a significant effect on all wing traits at both the plastic and evolutionary levels. When raised on poor larval food, both control and selected populations had smaller wings than when raised on standard food [62]. On the other hand, experiments with *Drosophila ananassae* showed that the level of fluctuating asymmetry was similar in flies reared on poor and standard media [54]. This means that drawing general conclusions based on the use of fluctuating asymmetry as an indicator of nutritional stress should be done with caution.

Symmetry is important for the movement of an animal, reveals its physiological state, and it also plays an important role in reproduction, courtship, and reproductive behavior [63,64,65]. The results of our research indicated that neomycin causes significant stress for larvae and imagoes of *D. melanogaster*. Both examined life stages revealed significant differences in the FA indices of measured parameters. The results obtained for larvae appeared to be concentration-dependent; the FA index increased with the concentration of neomycin. Interestingly, there was also a distinct difference between effects caused by lower (150 and 300 mg/L) and higher (600 and 900 mg/L) concentrations of the antibiotic. Although the lowest FA values were observed in the control group, low levels of antibiotics did not lead to a significant increase in asymmetry. The highest FA index, observed in the group exposed to 900 mg/L neomycin, showed that the larvae had a pronounced nutritional stress response. The Pearson correlation coefficients for anal papillae (0.65 and 0.71) indicated a correlation between neomycin concentration and asymmetry. The strong effect for larvae may be due to their softer cuticle, and the inability to avoid the exposed environment (the larvae cannot emigrate from the substratum). Following this stage, they undergo processes that lead to fast growth, and development of tissues and organs that prepare them for the pupal stage. Therefore, any stress may significantly affect their morphology. For measurements of the length of anal papillae, the results were inconclusive. Although the addition of neomycin reduced their size compared to the controls, which was consistent with our previous observations [3], there was no clear tendency to decrease in length as the concentration of the antibiotic increased. Interestingly, the lowest and highest concentrations did not cause the most distinct effects. It is possible that the low concentrations of the antibiotic did not stimulate the insects’ detoxifying pathways intensively enough, and neomycin remained in the body. On the other hand, the highest concentration might have been too high to be neutralized completely and some amounts of the antibiotic were still present in the environment.

The main role of the anal papillae is to regulate osmotic conditions. Neomycin has been described as a factor that affects concentration of potassium ions and blocks ryanodine RyR2 receptor [66,67,68]. The ryanodine receptors are also responsible for calcium storage within cells [69]. Therefore, due to altered concentrations of ions, this antibiotic may disturb osmotic conditions and, in consequence, lead to morphological malformations of anal papillae, which manifested in the measured FA. The ryanodine receptors are necessary for proper muscle physiology in fruit flies [70]. Hence, neomycin may significantly affect the development of insects and cause lethal and sublethal effects. These receptors are also present in insects’ photoreceptor cells, including in *Drosophila* [71]. However, to our best knowledge, the effect of the antibiotic on the physiology of *D. melanogaster* cuticle-forming epithelium and anal papillae, in particular, have not been reported to date. Therefore, we plan to carry out further studies on the ultrastructure and morphology of organs and tissues exposed to antibiotics in the future.

The wing FA indices indicated a large but variable correlation. The Pearson correlation coefficients varied from 0.52 to 0.87. However, there was no strict rule and the veins did not show the same pattern of the FA value alterations. On the other hand, the mean length of veins was the highest for the control group, with neomycin-exposed groups showing lower values. That suggests that the antibiotic may decrease the size of some morphological/anatomical organs. This result was in line with results obtained by Ventrella et al. [3] and Chowański et al. [4], where decreased size and shortened time of development of *D. melanogaster* exposed to some natural substances was observed. It is possible that the insects speed up their development to the imaginal stage in order to decrease the time of exposure to toxic substances. In consequence, some processes and metabolic pathways may malfunction; the growth may be decreased, which results in asymmetry and the limited size of morphological features. However, due to the longer period of development, the presence of the pupal stage (when the insect does not feed and reorganizes its internal structures), the ability to move away from the neomycin-containing substratum, and a stronger cuticle, the malformations may be less significant than in the case of larvae. Additionally, the process of detoxification may be more intensive in imagoes than in larvae. In consequence, FA indices were not as significant as in the case of larvae. However, asymmetrical veins may significantly affect flight ability in imagoes, since veins play a crucial role in wing strengthening and in conditioning the surface of wings. Therefore, the increased asymmetry may affect imagoes in their behavior, locomotion, and perhaps reproductive success.

Although not lethal in the tested range of concentrations, neomycin may cause significant sublethal changes that manifest in FA. In consequence, it may lead to lower vitality of flies exposed to the antibiotic and limit the population size. It is possible that similar results would be observed not only in our model species, but also in others. The harmful activity of neomycin on insects was previously reported for *Pimpla turionellae* [72]. Most probably, the effect is caused by reactive oxygen species (ROS), which are produced during exposure to neomycin and other aminoglycosides [73,74]. Additionally, an increased level of O_2_^−^ and H_2_O_2_, together with a decreased level of Cu, Zn superoxide dismutase was reported in cells exposed to gentamicin [75]. An imbalance between the level of ROS and antioxidant enzymes leads to oxidative stress within exposed organisms. If the cells are unable to achieve homeostasis, the imbalance will cause physiological abnormalities and morphological defects, which will lead to developmental errors and imperfections, which correlates with an increase in FA [76]. We plan to continue this research in the future, focusing on other antibiotics added to the media used to rear fruit flies, especially since *Drosophila melanogaster* is becoming increasingly recognized as a model organism in food and nutrition research.

## 5. Conclusions

Fluctuating asymmetry has been used as a bioinidicator of stress. In this paper, we measured FA in *D. melanogaster* larvae and imagoes reared on a neomycin-containing medium. Our findings showed that antibiotic may cause sublethal stress in *D. melanogaster* larvae and adults, and contribute significantly to morphological malformations of the exposed insects. This, in consequence, may lead to limited longevity, lower reproductive success, and decreased population size. The results suggest that neomycin, and perhaps other antibiotics, may cause significant physiological and developmental stress in insects and may possibly be used as insecticides in the future. However, such activity demands further research.

## Figures and Tables

**Table 1:** Correlation between two independent measurements (calculated from absolute values of differences between right and left anal papillae of *D. melanogaster* larvae), *** *p* < 0.001.

Anal Papillae	N	Pearson Correlation Coefficient	*p*-Value
AP1	49	0.65	<0.0001 ***
AP2	53	0.71	<0.0001 ***

**Table 2:** Result of ANOVA to detect asymmetry of anal papillae of *D. melanogaster* larvae, * *p* < 0.05, ** *p* < 0.01.

Anal Papillae	Mean of Longer Appendix	Mean of Shorter Appendix	AP1: F_(1,96)_ AP2: F_(1,104)_	*p*-Value
AP1	98.9	90.2	4.87	0.0297 *
AP2	63.8	56.9	11.10	0.0011 **

AP1—the first pair of anal papillae, AP2—the second pair of anal papillae.

**Table 3:** Correlation between two independent measurements (calculated on absolute values of differences between veins of right and left *D. melanogaster* wings).

Vein	N	Pearson Correlation Coefficient	*p*-Value
A	42	0.52	<0.0001
B	42	0.66	<0.0001
C	42	0.83	<0.0001
D	42	0.87	<0.0001
E	42	0.74	<0.0001
F	42	0.54	<0.0001

A–F—the measured veins, for their location see Figure 2; N—sample size.

**Table 4:** Result of ANOVA to detect asymmetry of *D. melanogaster* wing veins.

Vein	Mean of Longer Vein	Mean of Shorter Vein	F_(1, 82)_	*p*-Value
A	1352.6	1324.6	1.27	0.263
B	56.4	51.1	9.07	0.003 **
C	153.9	142.5	6.14	0.015 *
D	323.2	308.6	5.65	0.019 *
E	929.1	915.4	0.82	0.369
F	1386.5	1373.8	0.35	0.555

A–F—the measured veins, for their location see Figure 2.

**Table 5:** Descriptive statistics for FA for the anal papillae of *D. melanogaster* larvae.

Anal Papillae	Group	N	Mean	Min.	Max.	Range	SD	V%
AP 1	CA	12	0.05481	0.0089	0.1260	0.1170	0.039	70.56
	150	10	0.09347	0.0232	0.2046	0.1814	0.053	56.83
	300	9	0.08938	0.0085	0.1915	0.1831	0.072	80.27
	600	11	0.12023	0.0235	0.3109	0.2874	0.084	70.01
	900	7	0.19788	0.0116	0.3687	0.3570	0.144	72.54
AP 2	CA	9	0.06147	0.0039	0.1568	0.1529	0.053	86.15
	150	11	0.06655	0.0013	0.1927	0.1914	0.053	80.01
	300	13	0.08154	0.0032	0.1678	0.1646	0.052	64.30
	600	12	0.19467	0.0499	0.4492	0.3994	0.116	59.48
	900	8	0.22331	0.0867	0.4012	0.3145	0.098	43.77

ample size, Min—minimum value, Max—maximum value, SD—standard deviation, V%—coefficient of variability, CA—control group, AP 1—the first pair of anal papillae, AP 2—the second pair of anal papillae.

**Table 6:** Descriptive statistics for the three studied veins of *D. melanogaster* wings.

Vein	Group	N	Mean	Min.	Max.	Range	SD	V%
B	CA	12	0.09677	0.0104	0.2169	0.2065	0.055	56.88
	150	8	0.22371	0.0299	0.6875	0.6576	0.222	99.44
	300	7	0.06302	0.0233	0.1121	0.0889	0.033	52.27
	600	11	0.07752	0.0310	0.1374	0.1064	0.040	52.00
	900	4	0.13546	0.0164	0.2488	0.2325	0.107	79.35
C	CA	12	0.05021	0.0127	0.2807	0.2680	0.074	146.68
	150	8	0.16775	0.0147	0.9371	0.9224	0.315	187.71
	300	7	0.04436	0.0128	0.0889	0.0760	0.024	54.01
	600	11	0.08178	0.0219	0.2957	0.2738	0.083	101.79
	900	4	0.23740	0.0474	0.5152	0.4678	0.202	85.26
D	CA	12	0.04517	0.0214	0.0691	0.0477	0.015	32.49
	150	8	0.09850	0.0112	0.5013	0.4901	0.165	167.91
	300	7	0.03594	0.0137	0.0734	0.0598	0.025	68.86
	600	11	0.03677	0.0032	0.0691	0.0658	0.020	53.87
	900	4	0.01693	0.0089	0.0235	0.0146	0.007	38.47

N—sample size, Min—minimum value, Max—maximum value, SD—standard deviation, V%—coefficient of variability, CA—control group, B, C, D—studied veins.
